# A Memetic Algorithm for Solving the Robust Influence Maximization Problem on Complex Networks against Structural Failures

**DOI:** 10.3390/s22062191

**Published:** 2022-03-11

**Authors:** Delin Huang, Xiaojun Tan, Nanjie Chen, Zhengping Fan

**Affiliations:** School of Intelligent Systems Engineering, Sun Yat-sen University, Shenzhen 518107, China; huangdl5@mail2.sysu.edu.cn (D.H.); tanxj@mail.sysu.edu.cn (X.T.); chennj6@mail2.sysu.edu.cn (N.C.)

**Keywords:** complex networks, influence maximization, robustness, memetic algorithm, optimization

## Abstract

Many transport systems in the real world can be modeled as networked systems. Due to limited resources, only a few nodes can be selected as seeds in the system, whose role is to spread required information or control signals as widely as possible. This problem can be modeled as the influence maximization problem. Most of the existing selection strategies are based on the invariable network structure and have not touched upon the condition that the network is under structural failures. Related studies indicate that such strategies may not completely tackle complicated diffusion tasks in reality, and the robustness of the information diffusion process against perturbances is significant. To give a numerical performance criterion of seeds under structural failure, a measure has been developed to define the robust influence maximization (RIM) problem. Further, a memetic optimization algorithm (MA) which includes several problem-orientated operators to improve the search ability, termed ${\mathrm{RIM}}_{\mathrm{MA}}$, has been presented to deal with the RIM problem. Experimental results on synthetic networks and real-world networks validate the effectiveness of ${\mathrm{RIM}}_{\mathrm{MA}}$, its superiority over existing approaches is also shown.

## 1. Introduction

There are many networked systems in real life such as transportation networks and robot networks, which are indispensable parts of human work and life [1]. Automatic guided vehicles (AGVs), which belong to the category of wheeled mobile robots, play a significant role in transportation, the logistics industry, and autonomous driving [2], and can also be modeled as networked systems. The network topology information is widely studied for its direct description of the structural characteristics of systems. Some network structure characteristics including the random connection and the power law degree distribution, have been discovered and summarized in previous studies [3,4]. The network topology information plays a crucial role in related research and analysis takes on networked systems.

Due to the limited cost, resources cannot be allocated to all nodes in a network, but some influential nodes tend to be selected from the network as seeds to spread the influence. How to use the topological information of a specific network to select the seeds that can achieve the optimal propagation effect is defined as the influence maximization problem [5], which is of great significance in both theoretical and realistic applications. Applications of the influence maximization theory can be found in transportation networks such as the selection of cluster heads in the vehicular networks [6] and traffic bottleneck identification in the city [7].

For the influence propagation process in the networked systems, several information spreading models have been extensively emphasized, including the independent cascade (IC) model [8], the weighted cascade (WC) model [9], and the linear threshold (LT) model [10]. Kempe et al. modeled the seed selection problem as a discrete combinatorial optimization problem and proved the problem is NP-hard [8]. Another approach is through the Monte Carlo simulation, which directly estimates the influence range of seeds. The deficiency lies in the prohibitive computational cost; as the number of nodes enlarges, the required budget increases sharply. This method thus cannot be applied to large-scale networks. Lee et al. in [11] proposed a fast approximation method for influence spreading, and its rationality has been verified through experiments. The advantage is its lower computational cost. On the basis of these studies, the influence maximization problem can be regarded as an optimization problem, i.e., selecting the optimal set of nodes from the network guided by reasonable performance evaluation factors.

Networked systems are exposed to uncertainty and disturbances, and damages can even be destructive to the functionality. For the vehicular networks and robot networks, hacking smart terminals, disrupting cloud computing platform services, and cracking communication protocols are common forms of attack. According to the difference of attack targets, it can be divided into node-based and link-based attacks [12,13], both two categories have been demonstrated to be common and may cause serious damages. In terms of the attack type, it can be roughly divided into random attacks and malicious attacks. For the first category, targets in the network are attacked at the same probability. In malicious attacks, targets are attacked in the order of their importance; for example, nodes with larger degrees tend to be removed in priority [14]. Generally, malicious attacks are likely to cause more distinct structural losses than random attacks; therefore, such attack model has been intensively studied in previous studies including the attack on connectivity [15,16,17], on community [18,19], and on the diffusion behavior [13]. Several reasonable robustness performance evaluation factors have been designed [20,21]; based on these, methods that can improve the robustness are also developed [13,20,22,23,24].

Most of the existing studies only consider the situation that the network structure stays stable, and the selected seeds are only suitable for the current network structure [25,26,27,28]. Regarding the network-related influence maximization problem, there are some studies on how to robustly select seeds against potential uncertainties in the propagation process. These studies focus on the situation that the influence spreading probability or spreading model is uncertain [29,30]. Yet such factors have already been implied in the seed determination process, as shown in [5,7,9,25]. Meanwhile, the network structure is closely related to its performance. Changes in the network structure always impact the interaction between network nodes and further bring about disturbances on the influence propagation process. Consequently, the selection of seeds is expected to possess the ability to resist changes in the network structure and keep a relatively robust influential range. This important feature has not been thoroughly studied in the past literature. In other words, how to reasonably evaluate the influential performance of seeds when attacks on the network structure happen, and how to select the optimal seed set guided by the evaluation factor, these problems remain to be solved. Correspondingly, the robust influence maximization (RIM) problem is defined as the task of selecting a seed set that can maintain a good influence spreading ability under potential network structural damages.

Aiming at these deficiencies of the existing studies, this paper first analyzes how to robustly solve the problem of network influence maximization; the malicious attacks on networks are considered. Based on the robustness performance evaluation factors of the existing studies, a factor that evaluates the influence performance of the selected seeds under nodal attacks was designed, where a changeable parameter is included to control the damage extent. An experimental analysis was also conducted to determine a rational configuration of the parameter toward multiple scenarios. In this manner, the factor intuitively assesses the influence performance of seeds in a numerical form, and thus provides guidance for the optimal seed selection process. Equipped with which, a memetic algorithm is devised to select robust seeds under malicious node-based attacks. The proposed algorithm, ${\mathrm{RIM}}_{\mathrm{MA}}$, contains several problem-directed operators and exploits genetic information from both global and local areas. Corresponding experimental results on synthetic network and real network data indicate the superiority of ${\mathrm{RIM}}_{\mathrm{MA}}$ over existing methods. Meanwhile, tests are carried out on land transportation networks such as logistics networks and robot networks. The obtained seeds can achieve considerable influence performance when the network structure is under attack.

The rest of this paper is organized as follows. Section 2 represents the related works. Section 3 introduces the evaluation factor of robust influence performance proposed in this paper and the parametric configuration process. The ${\mathrm{RIM}}_{\mathrm{MA}}$ is described in detail in Section 4. Section 5 presents the experimental results and analysis. Finally, Section 6 summarizes this work and presents possible work.

## 2. Related Works

### 2.1. Influence Spreading Model and Evaluation Method

**Figure 1:**
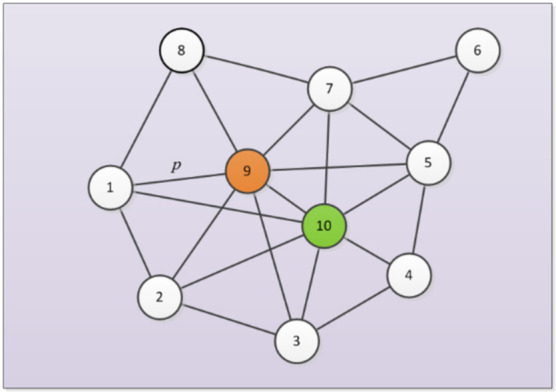
An example of the influence maximization on a simple network donated as G = (10, 22) which has 10 nodes and 22 edges. Node 9 and node 10 are the selected seeds that can generate the propagation maximally. In the IC model, node 9 has a fixed probability *p* to activate node 1.

A network can be modeled as a graph G = (V, E), where V = (1, 2, …, N) represents the set of N nodes, E = {$e_{ij}\;|$ i, j∈V, i≠j} represents the set of M edges between different nodes in the network. The influence maximization problem is to select the most influential *K* nodes from all nodes in the network as the seed set S = {$s_{1}$, $s_{2}$, …, $s_{k}$}, and the influential performance is donated as $\sigma \left(S\right)$ which is the maximum number of nodes that S can influence. A principle diagram of a simple network is shown in Figure 1. There are several existing spreading models to define the process of influence propagation process in the network. The widely-used spreading models include the IC model [8], the WC model [9], and the LT model [10], and slight differences can be found in the spreading rules. Taking the IC model as an example, nodes only have two states: active state or inactive state, and only the seed set S is active in the initialization phase. Details of the influence propagation process are as follows. At each time step t, the set of nodes that are active is donated as $S_{t}$, and $S_{0}=S$. Each node x in the $S_{t}$ only has one chance to activate each of its inactive connected neighbor nodes y at a pre-defined probability p at step t. Then, those successfully activated nodes are deposited into the temporary set $T_{t}$, the set of active nodes is updated as $S_{t+1}=S_{t}\cup T_{t}$. If the set $T_{t^{\prime }}\;$=∅, which indicates that there are no nodes activated at time step $t^{\prime }$, the process of influence propagation is terminated. $\sigma \left(S\right)$ is determined by the number of nodes in $S_{t^{\prime }}$. The difference between the WC model and IC model is that the activate probability between nodes is not definite, but is related to the weight information in the network. In the LT model, an inactive node is activated on the condition that the received total influence rate from neighboring nodes is larger than its predefined threshold. As shown in the simple network in Figure 1, node 9 and node 10 are selected as seeds to spread influence. In the initialization phase, only node 9 and node 10 are in an active state, while other nodes are in an inactive state. Taking the influence propagation process of node 9 as an example, in the IC model, node 9 has a fixed probability *p* to activate the surrounding nodes, while node 9 in the WC model has different probabilities to activate the surrounding nodes. The LT model is even more different. For example, the inactive state node 1 will only transform into the active state when the total influence rate of the three surrounding nodes reaches its pre-defined threshold. Considering that the IC model has been widely applied in existing studies [3,5,11], this work also employs this spreading model to investigate the robust influence maximization problem.

Given a spreading model, the Monte Carlo process is optional to evaluate the influence performance $\sigma \left(S\right)$ of the seed set S [8], but this method is time-consuming and may not get accurate estimation results. The specific process of the Monte Carlo simulation method to evaluate the influence of seeds is as follows. Assuming that the number of seeds is 10 and the number of simulations is *n* = 1000, the initial influence of the seeds $\sigma \left(S\right)$ is 10. Each simulation starts from the seeds and simulates whether each inactive node is activated under the probability p. If the node is activated, the seed influence $\sigma \left(S\right)$ is increased by 1, while the $\sigma \left(S\right)$ remains unchanged if the node is not activated. Then the final influence of the seeds recorded in the *n*-th simulation is $\sigma_{n}\left(S\right)$. This simulation is carried out 1000 times, sum and average all $\sigma_{n}\left(S\right)$ (divided by *n* = 1000), and the calculated average is the influence performance of the seeds. This method thus can only deal with evaluation tasks on small-scale networks. For improving the efficiency of the performance estimation process, Lee et al. in [11] proposed a fast approximation method for influence spreading; only the influence within the 2-hop range of seeds is considered, defined as follows:

(1) $$\hat{\sigma }\left(s\right)=\underset{s\in S}{\sum }\hat{\sigma }\left\{s\right\}-\left(\underset{s\in S}{\sum }\underset{c\in C_{s}\cap S}{\sum }p\left(s,c\right)\left(\sigma_{c}^{1}-p\left(c,s\right)\right)\right)-\chi$$

where $C_{s}$ is the 1-hop neighbor of node s, $p\left(s,c\right)$ is the propagation probability from active node s to inactive node c, χ represents the overlapped influence when the influence is estimated between two seeds. In Equation (1), the first term evaluates the initial 2-hop influence range of the seed node, the second term and the third term consider the 1-hop and 2-hop distances of the two seed nodes, respectively. The overlapped influence is to be subtracted. This fast approximation method can estimate the influence performance of seed reliably. Meanwhile, this method has the advantage of low computational cost and can tackle the evaluation task of seeds on large-scale networks.

### 2.2. Definition, Evaluation, and Optimization Methods of Network Structure Robustness

Most real-world networked systems are operated in open and complicated environments, and networks are threatened by unpredictable attacks and errors. Therefore, it is of great significance to study the robustness of networks. A robust networked system should be able to guarantee that its functionality keeps resistance against structural failures as much as possible. According to existing studies, an important indicator to evaluate the performance of networks is through the connectivity of the network structure.

A robustness evaluation factor R was proposed in [20] and R works as follows. Nodes in the network are sorted according to their importance based on certain criteria such as the degree of the node, these nodes are attacked in sequence. When a node is removed, the maximum connected cluster in the current network is recorded. The process does not terminate until the network is totally collapsed. The numerical evaluation result of the robustness of the network is through summation and normalization over the obtained cluster-evaluation results. The mathematical definition of R is

(2) $$R=\frac{1}{N}\overset{N}{\underset{Q=1}{\sum }}s\left(Q\right)$$

where N is the number of nodes in the network, $s\left(Q\right)$ is the proportion of nodes in the maximum connected cluster after removing Q nodes, and 1/N is a normalization factor, which guarantees that the comparison between networks with different sizes is achievable.

Zeng et al. in [31] made an extension on R, and an evaluation factor $R_{l}$ was designed to evaluate the network robustness under edge-based attacks. These factors including R and $R_{l}$ evaluate the robustness of networked systems from a numerical point of view, and can also guide the related optimization tasks. The evolutionary algorithm was employed to solve the optimization problem of network robustness optimization [13,22]. Although these network robustness evaluation factors cannot be directly applied to the robust influence maximization on networks, they provide references for the possible factor design process.

Intelligent vehicles and mobile robots, as part of land transportation systems, can be modeled as vehicular networks and robot networks because of the information interaction between nodes. Kim et al. in [32] modeled vehicle-to-vehicle information flow as a transportation network and proposed a diffusion framework for vehicular messaging. Basu et al. in [33] completed the fault-tolerant control to improve network topology according to the missing information of critical nodes in the robotic wireless communication network.

The influence maximization problem is also applied to land transportation networks. Wang et al. in [6] designed a cluster routing algorithm based on influence maximization, aiming to find the optimal cluster head of vehicles to improve the efficiency of the transmission. Zhao et al. in [7] modeled the traffic bottleneck identification problem as an influence maximization problem, and the goal is to find the most influential bottlenecks, which provides traffic planning solutions for decision makers. As a small land transportation system, wheeled mobile robots have shown significance in related studies [34,35] and can provide simplified models for subsequent research on larger land transportation systems.

## 3. Robust Influence Evaluation of Seeds under Network Structure Damage

In terms of the influence maximization problem, most studies ignore the impact of structural failures on the propagation ability of the selected seeds. In realistic applications, decision makers tend to be benefited by having robust seeds to deal with diversified situations. As mentioned, network attacks can be divided into node-based attacks and link-based ones. Attacks on nodes are direct and show effectiveness to collapse networks; as indicated by some previous studies [19,26], only the failure of a few nodes is enough to cause malfunction of the whole network. Considering the significance of nodes, this work concentrates on node-based attacks to investigate the robust influence performance.

### 3.1. Robust Influence Performance Evaluation Method

It has been proved that a malicious attack often causes greater losses to the structure and performance of networks. Under this circumstance, nodes are ranked according to their importance. The degree of nodes is a popularly-used measure to assess the importance. From the perspective of the attacker, the destruction operation is also limited by the available resources. If resources are sufficient, all nodes in the network can be destroyed; otherwise, only part of the nodes can be destroyed. Due to the significant structural importance, nodes with higher rank tend to be considered as priority in the destruction process.

In terms of the influence spreading, if a removal operation causes changes in the network structure, the maximal influence evaluation of the seeds should be re-estimated. Different network damage scenarios should be considered in the performance evaluation process; in this manner, the robustness of seeds can be guaranteed. Referring to Equation (2), the influence performance evaluation factor of seeds under node-based attacks is defined as follows:

(3) $$R_{s}=\frac{1}{N\times \rho }\overset{N\times \rho }{\underset{P=1}{\sum }}\hat{\sigma }\left(S|P\right)$$

where N is the number of nodes in the network. ρ is the ratio of attacked nodes. $\hat{\sigma }\left(S|P\right)$ is the estimated value of influence of the selected seed node set when P nodes are attacked. For a certain ρ, if the selected set S can obtain a larger $R_{s}$ value, it means that the selected set S is considered to be causing a greater influence under the current attack. Here, $1/\left(N\cdot \rho \right)$ works as the normalization factor.

### 3.2. Parameter Calibration in $R_{s}$

In network attacks, the degree of structural damage cannot be known in advance for decision makers, which makes it difficult to determine the parameter ρ. Once the parameter ρ in $R_{s}$ is determined, the factor can be used as an objective function to guide the seed selection process. Meanwhile, seeds obtained under different ρ are likely to be diverse. For example, when ρ is set as a small value, the selected seeds prefer the condition when the network stays steady or only suffers from slight damages. However, in the case of a larger value of ρ, the selected seeds prefer the condition that the network is severely damaged. Therefore, it is of great significance to select an appropriate ρ value so as to obtain a robust solution for possible scenarios.

**Figure 2:**
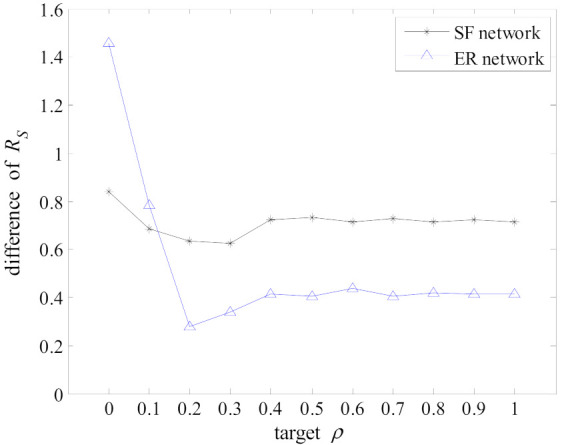
Numerical analyses of seeds’ performance guided by $R_{s}$ with different ρ on SF network and ER network. The results are averaged over 5 independent realizations.

In this subsection, a rational ρ value is determined through trials and errors. The experiments are conducted on two common synthetic networks, namely scale-free (SF) network [3] and random (ER) network [4], where the number of nodes is set as N=100 and the average degree is set as 〈k〉=4 [36]. The selection of seeds is carried out under the guidance of the factor $R_{s}$. The scale of seed set is configured as S=10, and the probability of spreading influence between nodes is set as p=0.01. The value of ρ is divided into 11 groups, lying in the range of [0,1] at an increasing step of 0.1, i.e., $\rho =\left\{0,\;0.1,\;0.2,\;\ldots ,\;1\right\}$. Seeds are selected guided by $R_{s}$ with different ρ values. It is necessary to verify the performance of the obtained seeds in the 11 groups. Each group of seed sets selected by a specific ρ is verified in 11 scenarios of different removal situations, i.e., 121 sets of tests in total. The difference between the specific result under a target ρ under each scenario and the optimal performance in this scenario is evaluated and summarized. If the sum of the differences is smaller, it can be considered that the selected seeds guided by the target ρ can achieve a relatively stable performance against multiple scenarios, indicating the seeds maintain better robustness against unknown attacks. The final experimental results are presented in Figure 2.

According to the experimental results in Figure 2, in the SF network, the seed set obtained when ρ is set to 0.2 and 0.3 can achieve a relatively stable propagation ability on all possible scenarios. In the ER network, the set of seeds selected under the condition of ρ=0.2 has the stable influence propagation ability in the test and shows distinct advantages over other configurations of ρ. In conclusion, the seed set selected when the ρ is 0.2 can achieve relatively stable influence propagation ability on two common artificial synthetic networks. Therefore, in the subsequent process of selecting seeds, ρ in $R_{s}$ is set as 0.2 to evaluate the performance of candidates.

## 4. RIM_MA_ Algorithm

**Figure 3:**
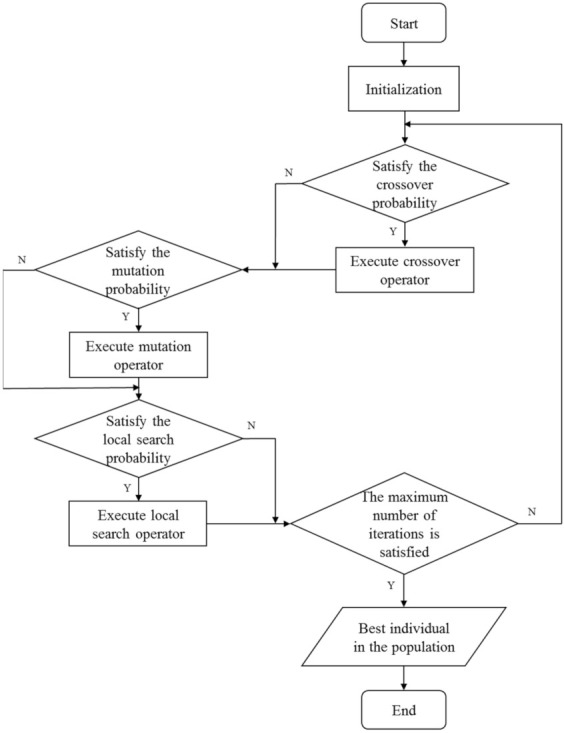
The procedure of the ${\mathrm{RIM}}_{\mathrm{MA}}$ algorithm.

In this section, operators of ${\mathrm{RIM}}_{\mathrm{MA}}$ are introduced in detail. The goal is to find candidates with the maximal robust influence factor $R_{s}$. In ${\mathrm{RIM}}_{\mathrm{MA}}$, $R_{s}$ is used as the fitness function to evaluate the quality of chromosomes. The procedure of the RIM algorithm is shown in Figure 3. First, an initialization operation is performed to obtain a random initial population. Then, randomly select different individuals from the population to execute the crossover operator to expand the population. The function of the mutation operator is to generate new individuals in the population to replace the old ones. Finally, the local search operator takes into account the local characteristics of nodes, and the target is to seek local replacement operations that can improve the fitness of the individual. After reaching the maximum number of iterations, the optimal individual in the population is output as the optimal solution. The details of each operator will be introduced in detail in each subsection. In the end, the framework of the ${\mathrm{RIM}}_{\mathrm{MA}}$ algorithm is summarized.

### 4.1. Initialization

**Figure 4:**
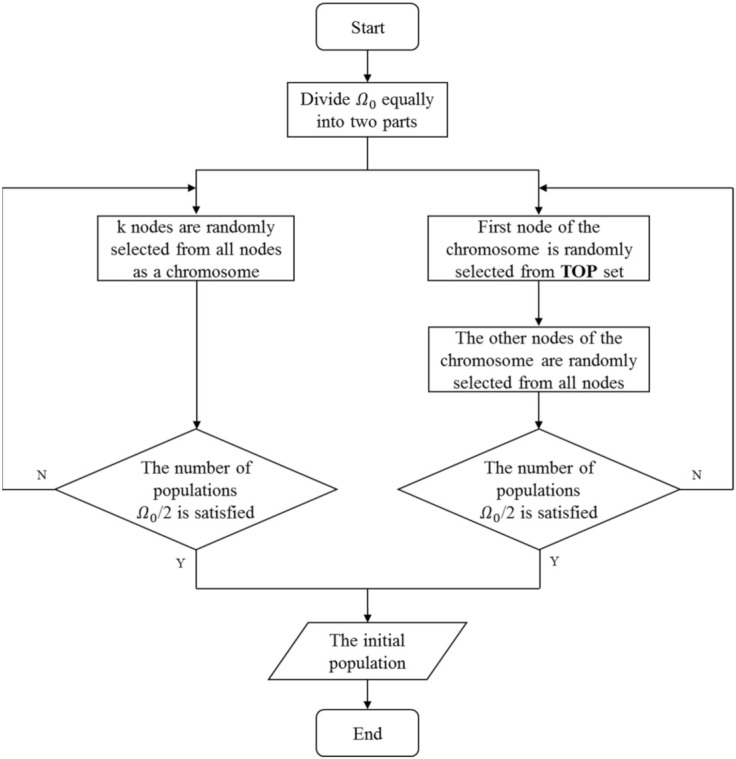
The procedure of the initialization algorithm.

In a memetic algorithm, each chromosome represents a set of seeds, which are the limited nodes selected to spread influence, and each individual contains a specific seed set. A population with Ω chromosomes represented Ω seed sets, which are labeled as $S_{1},S_{2},\ldots ,S_{\Omega }$. The initial population is generated by combining two strategies, i.e., random selection and degree preference selection. Specifically, the random selection strategy is adopted for the first half of the population. In detail, every chromosome selects *K* different seeds from *n* nodes in the network stochastically. The selection strategy of the other half population is based on the degree information of nodes. Nodes with a larger degree accounting for the top 2% in the network are preserved in the **TOP** set. The first node of the seed set of the individual is randomly selected from the **TOP** set, and the remaining nodes are randomly selected from other nodes in the network. Note that no duplicate seeds are allowed in a seed set; in that case, the duplicate one is replaced by a randomly generated seed. This strategy ensures that both nodes with high and low degrees are considered. The designed initialization operation is used to generate potential solutions widely distributed in the solution space, which is conducive to the subsequent optimization operations. The details of the crossover operator are summarized in Algorithm 1. The procedure of initialization is summarized as Figure 4. $P^{1}$ represents the initial population containing $\Omega_{0}$ individuals. $S_{i}^{1}$ represents the i-th individual in the population, and similarly $S_{k}^{1}$ represents the k-th individual.
**Algorithm 1.** Initialization**Input**:  $\Omega_{0}:$ Initial population size  k: Size of seed set;  G: Target network;**Output**:  $P^{1}=\left\{S_{1}^{1},S_{2}^{1},\ldots ,S_{\Omega_{0}}^{1}\right\}:$ Initial population;**for** i=1 **to** $\left(\Omega_{0}/2\right)$ do  **for** j=1 **to** k **do**    Randomly select a node from N nodes in G as the j-th element in the $S_{i}^{1}$    **while** (the j-th node in $S_{i}^{1}$ is the same as the rest) do      Randomly select a node from N nodes in G to replace j-th node    **end while**  **end for****end for****for** $k=\left(\Omega_{0}/2\right)$ **to** $\Omega_{0}$ **do**  The first node of $S_{k}^{1}$ is randomly selected from **TOP** set   **for** l=2 **to** k **do**    Randomly select a node from N nodes as the l-th element in the $S_{k}^{1}$  **end for****end for**

### 4.2. Crossover Operator

The purpose of the crossover operator is to exchange partial information between two chromosomes, and new chromosomes are generated to further enrich the current population. The crossover method adopted in this paper utilizes the single-point crossover at a probability of $p_{c}$. Assuming that $S_{p1}$ and $S_{p2}$ are two randomly selected parent chromosomes, an integer $L_{1}$ is randomly generated first in the range of $\left[1,K\right]$, and then the genetic information at $L_{1}$ in $S_{p1}$ and $S_{p2}$ is exchanged to generate two new child chromosomes, $S_{c1}$ and $S_{c2}$.

**Figure 5:**
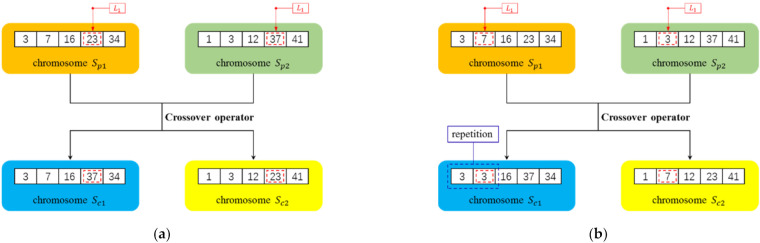
Examples for the crossover operator. The seed set size of the chromosome was set as 5. The red dotted box represents the crossover position and blue dotted box represents the position of repeated nodes. In (**a**), the crossover position $L_{1}$ was randomly selected as 4 to generate two legitimate child chromosomes and the crossover succeeded. In (**b**), $L_{1}$ was selected as 2 to generate illegitimate child chromosomes and the crossover failed.

**Figure 6:**
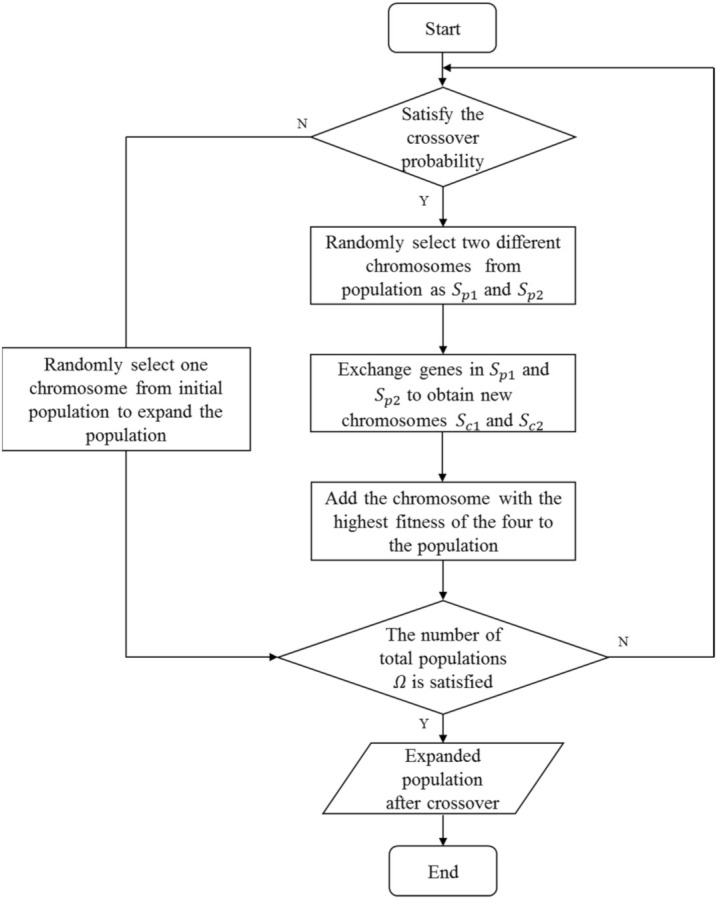
The procedure of crossover operator.

It should be noted that the randomly generated integer $L_{1}$ needs to ensure that the generated child chromosomes $S_{c1}$ and $S_{c2}$ are legitimate. In other words, the genetic information at $L_{1}$ in chromosome $S_{p1}$ cannot be duplicated with that in chromosome $S_{p2}$, and vice versa. If the random number $L_{1}$ cannot meet this condition, a new random number is generated. The details of the crossover operator are summarized in Algorithm 2. Examples of the operator are shown in Figure 5. The procedure of crossover operator is summarized as Figure 6. $S_{t}$ is a temporary set of two parent chromosomes and two child chromosomes. $S_{i}$ represents the i-th individual in the population.
**Algorithm 2.** Crossover**Input**:  $\Omega_{0}:$ Initial population size  Ω: Total population size  P: Current generation population  $p_{c}:$ Crossover probability;**Output**:  $P_{c}^{\prime }:$ Population after crossover;$P_{c}^{\prime }\leftarrow P$**for** $i=\left(\Omega_{0}+1\right)$ **to** Ω do  **if** $\left(r<p_{c}\right)$ /* r is a random number subjecting to uniform distribution between [0,1] */    Randomly select two different chromosomes from the population P as the parent chromosomes $S_{p1}$ and $S_{p2}$    Randomly generate an integer $L_{1}$ in the range of $\left[1,K\right]$    **while** (gene at $L_{1}$ on one parent chromosome is duplicated with the other) **do**      Randomly generate an integer $L_{1}$ again    **end while**    $S_{c1}\leftarrow S_{p1}$, $S_{c2}\leftarrow S_{p2}$    Remove the node at $L_{1}$ from $S_{c1}$ and add the node at $L_{1}$ from $S_{p2}$ to $S_{c1}$    Remove the node at $L_{1}$ from $S_{c2}$ and add the node at $L_{1}$ from $S_{p1}$ to $S_{c2}$    Calculate the fitness of $S_{p1}$, $S_{p2}$, $S_{c1}$, $S_{c2}$, and the chromosome with the largest fitness is denoted as $S_{t}$    $S_{i}\leftarrow S_{t}$    add $S_{i}$ to $P_{c}^{\prime }$  **else**    randomly select a chromosome $S_{t}$ from P    $S_{i}\leftarrow S_{t}$    add $S_{i}$ to $P_{c}^{\prime }$  **end if****end for**Output the expanded population $P_{c}^{\prime }$;

### 4.3. Mutation Operator

**Figure 7:**
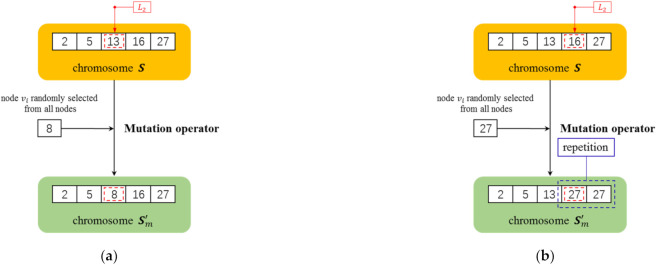
Examples for the mutation operator. The seed set size of the chromosome was set as 5. The red dotted box represents the mutation position and blue dotted box represents the position of repeated nodes. In (**a**), the mutation position $L_{2}$ was randomly selected as 3 to generate legitimate chromosomes and the mutation succeeded. In (**b**), $L_{2}$ was selected as 4 to generate illegitimate chromosomes and the mutation failed.

**Figure 8:**
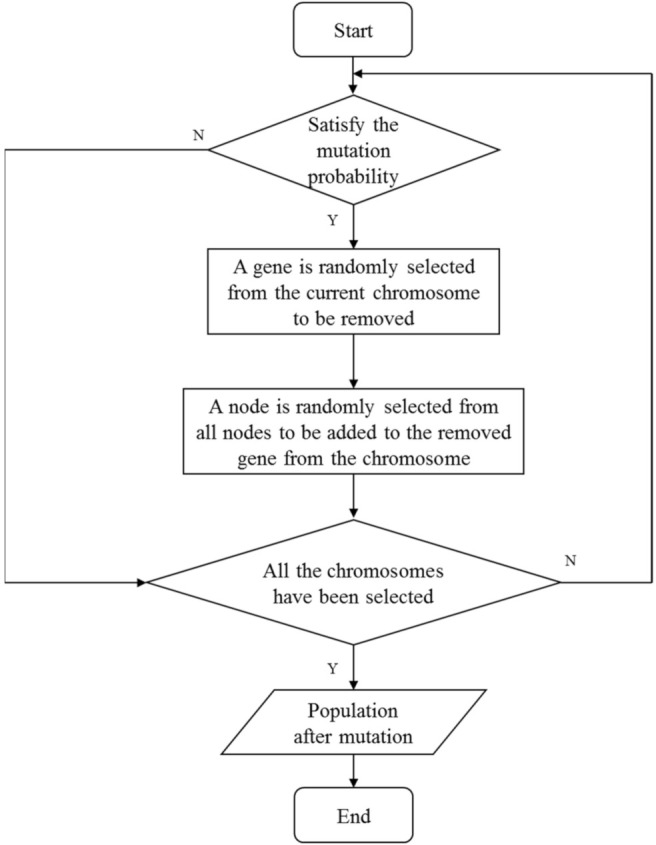
The procedure of mutation operator.

For all chromosomes in the population, the mutation operator is performed at a probability of $p_{m}$. Similar to the crossover operation, for the chromosome S to be mutated, an integer $L_{2}$ in the range of $\left[1,K\right]$ is randomly generated, and then a node is randomly selected from all N nodes to replace the gene at $L_{2}$ in the chromosome S. Similarly, in order to ensure that the mutated chromosome $S_{m}^{\prime }$ is legitimate, it is necessary to guarantee that the selected replacement node is not duplicated with all nodes in S; otherwise, the replacement node should be selected again until it meets the condition. The details of the mutation operator are given in the following Algorithm 3. Examples of the mutation operator are shown in Figure 7. The procedure of mutation operator is summarized as Figure 8.


**Algorithm 3.** Mutation
**Input:**
  P: Population before mutation  $p_{m}:$ Mutation probability;
**Output:**
  $P_{m}^{\prime }:$ Population after mutation;

$P_{m}^{\prime }\leftarrow P$

**for** (each chromosome $S_{i}$ in $P_{m}^{\prime }$) **do**  **if**
$\left(r<p_{m}\right)$ /* r is a random number subjecting to uniform distribution between [0,1] */   Randomly generate an integer $L_{2}$ in the range of $\left[1,K\right]$   Randomly select a node $v_{t}$ from all N nodes   **while** ($v_{t}$ is duplicated with all nodes in $S_{i}$) **do**     Randomly select a node $v_{t}$ from all N nodes again   **end while**   Remove the node at $L_{2}$ from $S_{i}$ and add the node $v_{t}$ to $S_{i}$  **end if**
**end for**



### 4.4. Local Search Operator

The local search operator is an important operation that distinguishes MA from GA. Two strategies are considered in the operator. Firstly, the operator should consider the local characteristics of the node such as its 2-hop neighborhood. Secondly, those nodes with larger degrees are preferred in the early stage of the algorithm, which is aimed at promoting the fitness function. As the iteration time increases, the probability of such operations is reduced to avoid premature convergence.

**Figure 9:**
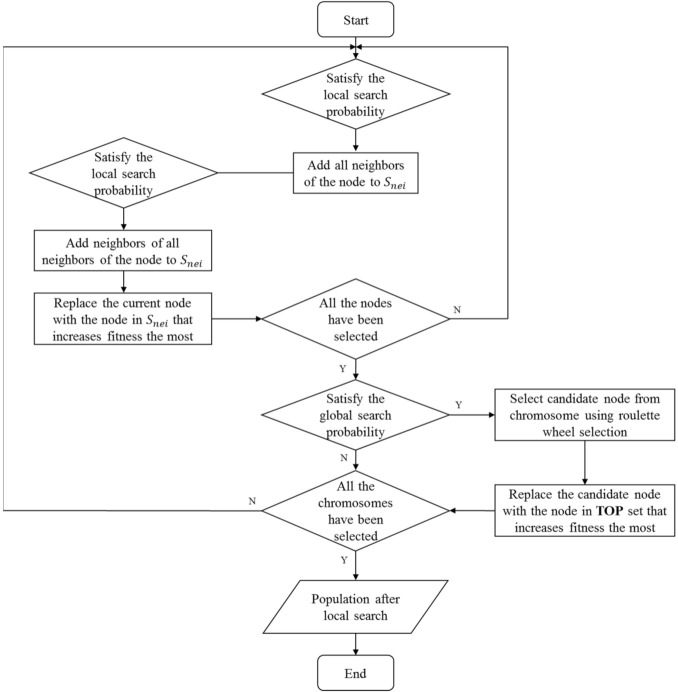
The procedure of local search operator.

Based on the above strategy, the local search operator is divided into two phases. The first phase is the local search toward the nodal neighborhood, which is performed at a probability of $p_{mi}$. For each seed in the chromosome, its neighbors or neighbors’ neighbors are searched to find better candidates. In order to limit the computational cost, this 2-hop replacement is only performed at a small probability. The fitness of the replaced seed set is evaluated, and only the operations that reach a better performance are retained. The second part is the global search of nodes, which is performed at a varying probability, and the probability decreases as the number of iterations increases. Applying the roulette wheel selection, the node that is to be replaced in the current chromosome is selected. The strategy of roulette wheel selection is based on the degree of seed. The smaller the degree, the higher the probability of being selected. Nodes with smaller degrees may also be important nodes in the network, so all nodes should have a chance of being selected. The operation is inclined to replace those low-degree ones in priority. If the performance of this seed set gets promoted, then the replacement operation is kept. The specific details of the local search operator are given in Algorithm 4. The procedure of local search operator is summarized as Figure 9. $S_{Nei}$ represents the temporary set of 1-hop and 2-hop of the node. $s_{l}$ represents the replacement node in $S_{Nei}$ that can improve the individual performance the most. $s_{g}$ represents the replacement node in the **TOP** set that can improve the individual performance the most.
**Algorithm 4.** Local Search**Input**:  P: Current generation population  $p_{mi}:$ Local search probability  $p_{ma}:$ Global search probability  gen: Current iteration  MaxGen: Maximum iterations;**Output**:  $P_{l}^{\prime }:$ Population after local search;$P_{l}^{\prime }\leftarrow P$**for** (each chromosome $S_{i}$ in $P_{l}^{\prime }$) **do**  **for** (each seed s in $S_{i}$) **do**    **if** $\left(r<p_{mi}\right)$ /* r is a random number subjecting to uniform distribution between [0,1] */      **for** (each neighbor node $s_{n}$ of s) **do**        Add $s_{n}$ into the set $S_{Nei}$        **for** (each neighbor node $s_{nn}$ of $s_{n}$) **do**          **if** $\left(r<p_{mi}\right)$           Add $s_{nn}$ into the set $S_{Nei}$          **end if**        **end for**     **end for**     Try to replace s with each node in the set $S_{Nei}$. If the fitness is improved, the neighbor node with the largest fitness is recorded as $s_{l}$     Remove s and add $s_{l}$ into $S_{i}$   **end if**   **if** $\left(r<\left(p_{ma}\times \left(MaxGen-gen\right)/MaxGen\right)\right)$     Obtain the **TOP** set of nodes with a large degree accounting for 2% of the total nodes;     Select a node s from $S_{i}$ using roulette wheel selection     /* The smaller the degree, the higher the probability of being selected */     Try to replace s with each node in the **TOP** set. If the fitness is improved, the node with the largest fitness is recorded as $s_{g}$     Remove s and add $s_{g}$ into $S_{i}$   **end if**  **end for****end for**

### 4.5. **RIM_MA_** Framework

In ${\mathrm{RIM}}_{\mathrm{MA}}$, the initialization operator is performed first to obtain the initial population. In each generation of the ${\mathrm{RIM}}_{\mathrm{MA}}$, the crossover operator is performed to enrich the population; then the mutation operator is performed. Followed by the local search operator, the fitness level of the whole population is to be promoted. At the end of each iteration, the fitness function of each chromosome in the population is evaluated, and the best individual is preserved into the next population, while other individuals are selected from individuals in the current population based on a roulette wheel selection according to their fitness. The higher the fitness, the greater the probability of being selected. The above process is repeated until the iterations reach the pre-defined threshold, then the overall best candidate is the final output. The overall framework of ${\mathrm{RIM}}_{\mathrm{MA}}$ is summarized in Algorithm 5.
**Algorithm 5.**${\mathrm{RIM}}_{\mathrm{MA}}$**Input**:  G: Target network  $\Omega_{0}:$ Initial population size  Ω: Total population size  k: Size of seed set  $p_{c}:$ Crossover probability  $p_{m}:$ Mutation probability  $p_{mi}:$ Local search probability  $p_{ma}:$ Global search probability  MaxGen: Maximum iterations;**Output**:  $S^{*}=\left\{s_{1},s_{2},\ldots ,s_{k}\right\}:$ Optimal seed set;$P^{1}=\left\{S_{1}^{1},S_{2}^{1},\ldots ,S_{\Omega_{0}}^{1}\right\}\leftarrow$Initialization$\left(G,\Omega_{0},k\right)$**for** g=1 to MaxGen **do**  $P_{t}\leftarrow \emptyset$  **Repeat**    Randomly select two different chromosomes from the population $P^{g}$ as the parent chromosomes $S_{pi}$ and $S_{pj}$    ($S_{ci},S_{cj}$)←Crossover ($S_{pi},S_{pj},p_{c}$)    $P_{t}\leftarrow P_{t}\cup \left(S_{ci},S_{cj}\right)$
  **Until** (all chromosomes in $P^{g}$ have been selected)  **for** (each chromosome S in $P_{t}$ and $P^{g}$) **do**    $P_{m}^{g}\leftarrow$Mutation (S,$\;p_{m}$)  **end for**  **for** (each chromosome $S_{m}$ in $P_{m}^{g}$) **do**    $P_{l}^{g}\leftarrow$Local_Search ($S_{m}$,$\;p_{mi},p_{ma}$)  **end for**  $P^{g+1}\leftarrow$Selection_Operator ($P_{l}^{g}$);**end for**Output the current best individual;

## 5. Experiments

In order to verify the performance of the designed ${\mathrm{RIM}}_{\mathrm{MA}}$, experiments on three synthetic networks are conducted first, including scale-free (SF) networks [3], random (ER) networks [4], and small-world (SW) networks [37], and then those on realistic networks are presented. In this paper, we implement the comparison of various existing seed selection algorithms such as simplified memetic algorithm (MA-sim), genetic algorithm (GA), simulated annealing algorithm (SAA), and degree-based algorithm (DBA) with the ${\mathrm{RIM}}_{\mathrm{MA}}$. The factor $R_{s}$ in Equation (2) is used to evaluate the performance of seeds selected by algorithms. Further, experiments are conducted on several land transportation networks to validate the effectiveness of the developed algorithm.

To compare the Monte Carlo simulation method and the 2-hop fast approximation method, we also conduct experiments with a small-scale network. The above two methods are used to evaluate the influence of the seeds obtained by the four algorithms. The numerical values and computation time of the two evaluation methods are shown in Table 1. $\sigma \left(S\right)$ represents the influence of seeds evaluated using the Monte Carlo simulation method. $\hat{\sigma }\left(S\right)$ represents the influence of seeds evaluated using the 2-hop fast approximation method. There is almost no difference in the performance of the two evaluation methods, but the computation time of the Monte Carlo simulation method is more than ten times that of the 2-hop fast approximation method. This also shows that the Monte Carlo simulation method is not suitable as the fitness function of the evolutionary algorithm, and this method cannot tackle the evaluation task on networks of a large scale.

**Figure 10:**
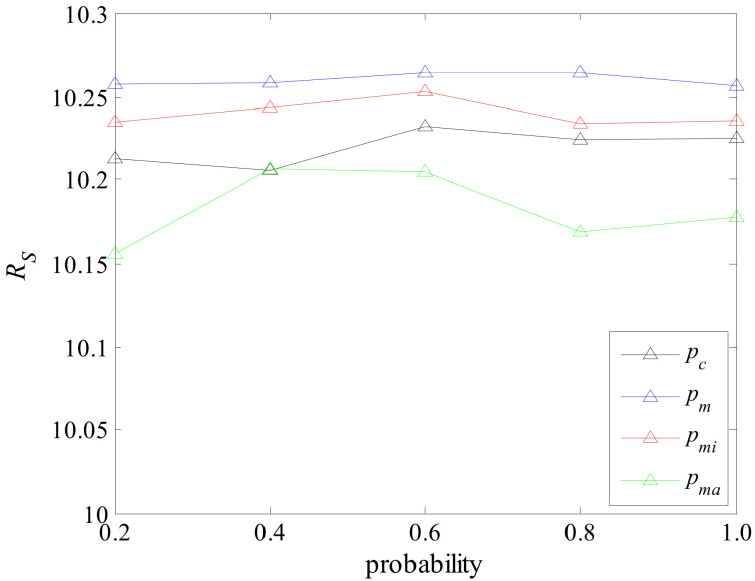
Performance of the algorithm with different parameters.

The various parameters of ${\mathrm{RIM}}_{\mathrm{MA}}$ are set as follows. The maximum number of iterations MaxGen is 150, the size of population Ω is 50. We conducted a simple experiment to determine the parameters $p_{c}$, $p_{m}$, $p_{mi}$, $p_{ma}$. The experimental method is as follows. In the same network, only the test parameters are changed and other parameters remain unchanged to test the performance of the algorithm. The final experimental results are shown in Figure 10. According to the results in Figure 10, when the parameter $p_{c}$ is set to 0.6, the performance of the algorithm is better than when $p_{c}$ is set to the other four values, and the same is true for $p_{m}$ and $p_{mi}$. Additionally, when $p_{ma}$ is set to 0.4, the performance of the algorithm is better than setting the other four values. Therefore, we set $p_{c}$, $p_{m}$, $p_{mi}$ to 0.6, and $p_{ma}$ to 0.4. In order to ensure comparability, GA also uses similar parameters. The parameters of MA-sim are consistent with the ${\mathrm{RIM}}_{\mathrm{MA}}$. The only difference is that the local search of the node neighborhood is omitted from the local search operator, and only the global search of the node is retained.

### 5.1. Experiments on the Synthetic Networks

SF networks, ER networks, and WS networks with different scales are generated to compare the proposed ${\mathrm{RIM}}_{\mathrm{MA}}$ with other existing algorithms in this experiment. The experiments are conducted on three artificial synthetic networks with 100, 300, 500, and 1000 nodes $\left(N\right)$ where the average degree of the network is set to 4. Results of each algorithm in a specific network are averaged over 20 independent realizations. As aforementioned, the parameter ρ of the factor $R_{s}$ is set to 0.2. The specific value of the influence maximization performance quantitative index $R_{s}$ of the seed set is given in Table 2.

It can be seen that in three artificial synthesis networks with different scales, the seed set selected by all evolutionary algorithms including ${\mathrm{RIM}}_{\mathrm{MA}}$, MA-sim, and GA achieve a higher $R_{s}$ value than the seed set obtained by other algorithms. This phenomenon indicates that these seed sets have a better ability to spread influence under uncertain deliberate attacks. The seed sets selected by the degree-based algorithm have the worst performance of influence propagation. It also shows that only relying on the information of the original network such as the degree is inadequate, and the obtained seed set often cannot cope with the network structure damage when the network is attacked. Specifically, the degree-based algorithm preferentially selects the nodes with a higher degree of network, while the malicious attack also preferentially selects these nodes. When the number of attacked nodes is large, all the seeds have been attacked and cannot spread influence. When the number of attacked nodes is small, only a few seeds in the network can still spread influence. Therefore, the seed set selected by the degree-based algorithm scores a low $R_{s}$, and such seeds may not tackle the robust influence maximization task.

**Figure 11:**
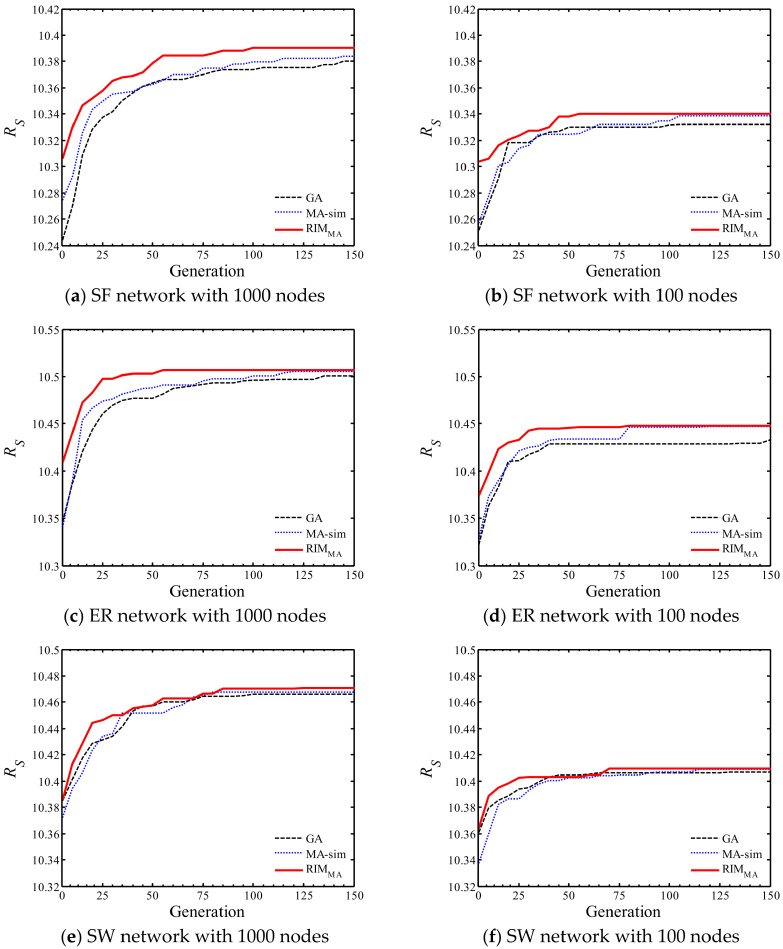
The evolution process of three evolutionary algorithms on (**a**) large-scale SF network, (**b**) small-scale SF network, (**c**) large-scale ER network, (**d**) small-scale ER network, (**e**) large-scale SW network, (**f**) small-scale SW network.

**Figure 12:**
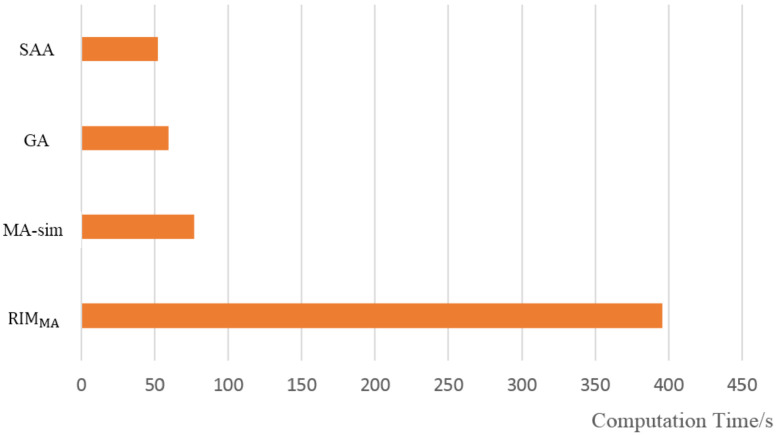
Result of computation time for the four algorithms in SF network with 100 nodes.

Comparing the three evolutionary algorithms, the ${\mathrm{RIM}}_{\mathrm{MA}}$ tends to achieve better results, and seeds selected by ${\mathrm{RIM}}_{\mathrm{MA}}$ can obtain the best influence propagation performance when the network is attacked. MA-sim and GA are inferior. It is worth mentioning that in the SW network with 100 nodes, the influence propagation performance of the seed set selected by the MA-sim is slightly better than that of the seed set selected by the ${\mathrm{RIM}}_{\mathrm{MA}}$, which may be caused by the small scale of the network and the unique neighboring connected structure of SW networks. The convergence process of the three evolutionary algorithms is further analyzed. Figure 11 shows the convergence curves of the three algorithms on SF networks with different scales. For large-scale or small-scale SF networks and ER networks, the performance of the initial seed set selected by ${\mathrm{RIM}}_{\mathrm{MA}}$ is significantly better than the sets selected by the other two evolutionary algorithms due to the addition of diversified structural information in the initialization, and ${\mathrm{RIM}}_{\mathrm{MA}}$ maintains superiority over the whole evolution process. The advantage of ${\mathrm{RIM}}_{\mathrm{MA}}$ is not so marked compared with other networks on SW networks, but the algorithm is still effective for selecting powerful seeds. In general, the experimental results verify that the ${\mathrm{RIM}}_{\mathrm{MA}}$ can be well applied to common synthetic networks with different scales, and the generality is considerable. The computation time of each algorithm is shown in Figure 12 compared with the other three algorithms, ${\mathrm{RIM}}_{\mathrm{MA}}$ is computationally expensive. However, the experiments prove that these excess costs are reasonable, and ${\mathrm{RIM}}_{\mathrm{MA}}$ provides a more competitive solution for decision makers.

### 5.2. Experiments on the Realistic Land Transportation Networks

In order to further verify the performance of the algorithm, two real-world networks are selected in this section, and the above five algorithms are used to select seed sets to make comparisons. The first is a logistics transport network in a certain area of Berlin, denoted as $G_{B}$ [38]. This network consists of 224 nodes and 376 edges, where each node represents a freight station and each edge represents a viable transportation route between freight stations.

**Figure 13:**
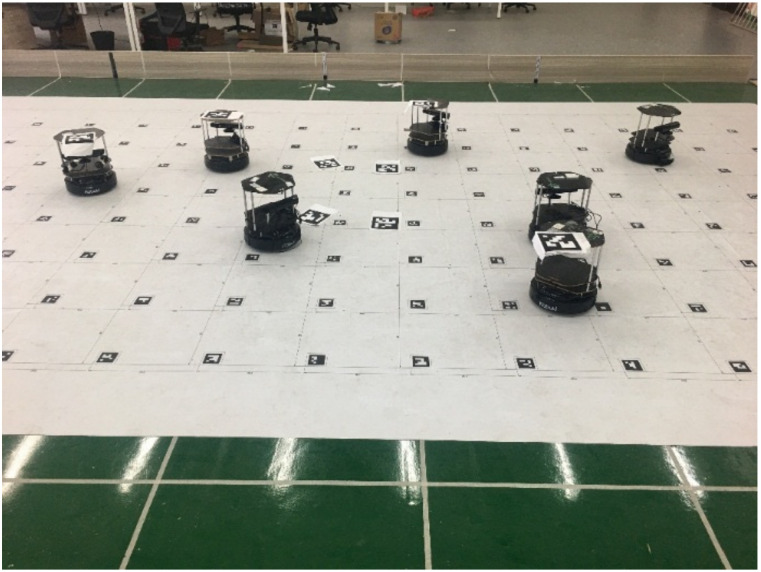
Wheel mobile robots (TurtleBot2) in Sun Yat-sen University.

The second is a larger-scale robot network based on the existing robots in Sun Yat-sen University as shown in Figure 13. According to the distribution, it can be divided into two types: random distribution and cluster distribution. The random robot network is denoted as $G_{R1}$ and the cluster robot network as $G_{R2}$, which both consist of 200 nodes. In these networks, each node represents a robot, and each edge represents the communication between robots. Different from other networks, the communication between nodes in a robot network is closely related to the distance between nodes. In other words, due to physical equipment, two robots cannot communicate with each other when they are far apart unless the distance between two robots is within a threshold range.

**Figure 14:**
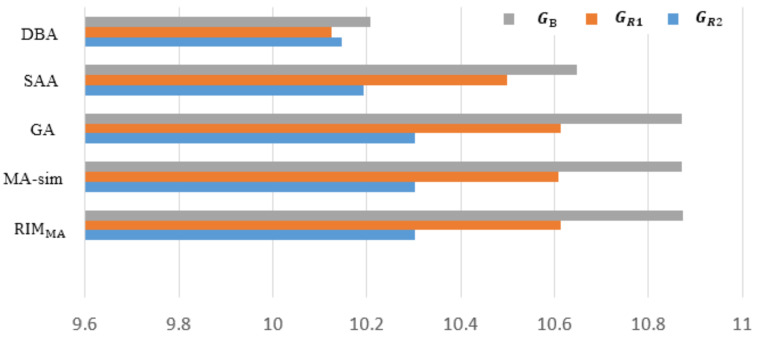
The $R_{s}$ performance comparison of five algorithms on three networks.

**Figure 15:**
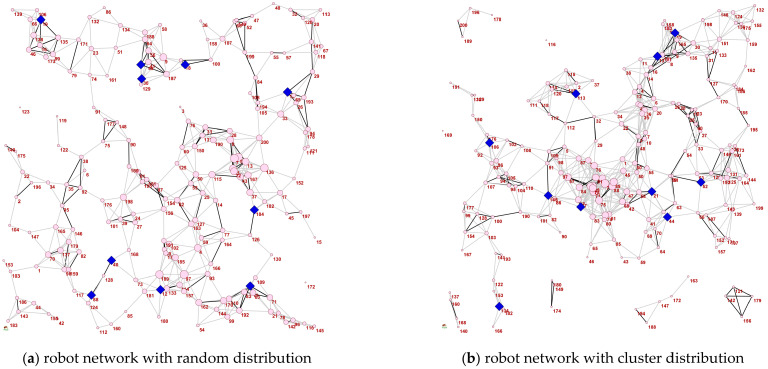
The topologies of robot networks and seeds selected by ${\mathrm{RIM}}_{\mathrm{MA}}$.

Figure 14 shows the experimental results of tested algorithms on three realistic networked systems. More specific performance of each algorithm is shown in Table 3. It can be concluded that ${\mathrm{RIM}}_{\mathrm{MA}}$ is also superior over the other four algorithms in the experiment, and seeds selected by the algorithm can achieve the maximum propagation when the network is under attack. Note that the performance of GA is better than MA-sim in $G_{R1}$ and $G_{R2}$. This may be due to the particularity of the robot network connection, which limits the effectiveness of the global search operator in MA-sim. The local search operator in ${\mathrm{RIM}}_{\mathrm{MA}}$ can effectively find seeds with considerable propagation performance. Figure 15 shows the topological structure of $G_{R1}$ and $G_{R2}$ robot networks, in which the blue diamonds represent the selected seeds, from which we can see the structural characteristics of seeds with robust influence ability. One feature is that the degree of seeds is relatively smooth. If two seeds are closely connected, the generated influence may be overlapped, which tends to cause duplicated transmission resources. Due to the limited number of seeds, inactive seeds in other areas may not be handled. Secondly, the proportion of seeds with a large degree is small. As the target of malicious attack is to remove hubs in the network first, such nodes thus cannot achieve the spreading task and the obtained $R_{s}$ value tends to be inferior.

Experiments on three realistic networks further demonstrate the effectiveness of ${\mathrm{RIM}}_{\mathrm{MA}}$, and also reveal that the proposed algorithm can provide some countermeasures for decision makers to solve realistic problems. For the Berlin logistics network, robust influential seeds can serve as an alternative solution to improve the overall transportation efficiency when attacks happen. For the robot networks, the seeds selected from the network are generally the key robots. These robots are crucial to complete the communication and information interaction tasks between robots under situations such as structural attacks and other emergencies. In summary, the ${\mathrm{RIM}}_{\mathrm{MA}}$ designed in this paper can effectively solve the problem of robust influence maximization, whether for some common artificial synthetic networks of different scales or some actual networks. On the other hand, the significance of determining seeds with robust influence ability is also shown in some real-world systems.

## 6. Conclusions

In this paper, based on the existing research on network influence maximization, the concept of robustness was introduced, and the problem of robust influence maximization was defined. Considering the challenges and problems in the land transportation network, the selection strategy on critical nodes under structural damage was studied. Both the information diffusion process and structural perturbances were considered, and the ultimate goal was to find seeds with robust influence ability against structural damages. Firstly, the IC model was adopted to simulate the diffusion process, and the 2-hop influential range of seeds was concentrated. Referring to the existing literature, an evaluation factor was designed to numerically evaluate the influence propagation performance of seeds under attacks. Then, ${\mathrm{RIM}}_{\mathrm{MA}}$ was designed to solve the robust influence maximization problem. The algorithm fully considers the optimal information from both neighboring and global areas, and the seed set with the maximal robust influence in the network is expected. Finally, experimental results on synthetic networks and realistic networks revealed that the performance of ${\mathrm{RIM}}_{\mathrm{MA}}$ is competitive compared with existing algorithms, valuable candidates are obtained for decision makers. The results provide references for solving problems such as knowledge mining, system control, and emergency management of several networks, which contribute to the development and application of land transportation systems.

In the future, there are still several difficult problems that can be further studied. First of all, the propagation model employed in this paper is the IC model. More propagation models such as the WC model and the LT model are to be studied. Then, many parameters in the experiments are configured as fixed values, including the damage ratio  ρ in the measure $R_{s}$. Consequent studies on the problem of RIM with uncertain parameters are desired. Finally, how to solve the problem of robust influence maximization in complicated systems such as the multiplex network [39] and the interdependent network [40] are also worthy of further investigations.

## Figures and Tables

**Table 1:** Differences between the two influence evaluation methods.

INDEX	$RIM_{MA}$	GA	SAA	DBA
$\sigma \left(S\right)$/time (s)	10.341/2.3 s	10.338/2.3 s	10.276/2.3 s	10.125/2.3 s
$\hat{\sigma }\left(S\right)$/time (s)	10.340/0.2 s	10.337/0.2 s	10.276/0.2 s	10.124/0.2 s

**Table 2:** $R_{s}$ performance comparison of seeds selected by different algorithms on three synthetic networks with different scales.

NETWORK	*N*	*RIM_MA_*	*MA-SIM*	*GA*	*SAA*	*DBA*
SF	100	**10.33951**	10.33763	10.33724	10.27430	10.12350
	300	**10.39330**	10.39202	10.38694	10.30001	10.07035
	500	**10.37831**	10.37818	10.37654	10.28429	10.06847
	1000	**10.38623**	10.38380	10.38086	10.27163	10.04240
ER	100	**10.44711**	10.44512	10.44495	10.36656	10.14899
	300	**10.49732**	10.49529	10.49528	10.38567	10.08195
	500	**10.50606**	10.50525	10.50349	10.39711	10.07562
	1000	**10.51805**	10.51729	10.51593	10.38548	10.02223
SW	100	10.40811	**10.40824**	10.40782	10.35068	10.13332
	300	**10.43799**	10.43788	10.43650	10.36159	10.04350
	500	**10.44381**	10.44228	10.44194	10.38061	10.04867
	1000	**10.46810**	10.46741	10.46730	10.38103	10.10572

**Table 3:** $R_{s}$ performance comparison of seeds selected by different algorithms on three realistic networks.

NETWORK	*N*	RIM_MA_	MA-SIM	GA	SAA	DBA
***G_B_***	224	**10.30328**	10.30262	10.30187	10.19373	10.14699
***G_R_*_1_**	200	**10.61319**	10.60826	10.61282	10.49883	10.12651
***G_R_*_2_**	200	**10.87442**	10.87051	10.87071	10.64649	10.20853

## Data Availability

Not applicable.
